# Perceived effectiveness of graphic health warnings as a deterrent for smoking initiation among adolescents in selected schools in southwest Nigeria

**DOI:** 10.1186/s12971-016-0074-y

**Published:** 2016-03-18

**Authors:** A. O. Adebiyi, O. C. Uchendu, E. Bamgboye, O. Ibitoye, B. Omotola

**Affiliations:** Department of Preventive Medicine and Primary Care, University of Ibadan, Ibadan, Nigeria; Department of Community Medicine, University College Hospital, Ibadan, Nigeria; College of Medicine, University of Ibadan, Ibadan, Nigeria

**Keywords:** Graphic health warning, Tobacco, FCTC, Smoking

## Abstract

**Background:**

There has been a sustained increment in young people initiating smoking in low middle income countries like Nigeria. Health warnings on cigarette packages are a prominent source of health information and an effective means of communicating specific disease risks to adolescents and young adults alike. This study evaluated the perceived effectiveness of selected graphic warnings on smoking initiation amongst in-school adolescents.

**Methods:**

This was a cross-sectional study conducted amongst secondary school students aged 13–17years in Igbo-Ora, Nigeria. A two-stage sampling technique with the school classes as the final sampling unit was used to select the students. An interviewer assisted questionnaire was used to obtain information on students demographic characteristics and their perception of graphic warnings using four images from the pictorial health warning galleries of the World Health Organization showing: ‘cigarette smoking causes cancer of the airways, harms children, causes stroke and causes impotence respectively'.

**Results:**

A total of 544 senior secondary students were included in this study with a male female ratio of 0.8:1. Of those interviewed, 40 (7.4 %) indicated that they had ever considered smoking, nine (1.7 %) responded that they had ever smoked and two students indicated that they were current smokers.

With all the images, fear was the dominant emotion expressed by the respondents. This was expressed by 307 (56.4), 215 (39.5), 203 (37.3) and 228 (41.9 %) respondents to images 1, 2, 3, and 4 respectively. Furthermore, 76.7, 44.7, 58.5 and 62.1 % of respondents felt Images 1, 2, 3 and 4 respectively will to a large extent prevent people from initiating smoking. There was no association between perceived effectiveness and gender. However, those younger than 15 years rated images on cancer of the airway and impotence as probably effective to a larger extent than did those who were 15 years and older (*p* = 0.032).

**Conclusion:**

Introduction of graphic health warnings, especially with an imagery depicting cancer and impotence may influence non-smokers to remain abstinent. Therefore, this study provides a template for a future policy-relevant study on graphic health warning in Nigeria.

## Background

The sustained drive to reduce tobacco consumption in high and upper middle income countries has shifted the focus of the Tobacco Industry to Africa and other Low and Middle Income Countries (LMICs). For instance, between 1990 and 2009, cigarette use dropped by 26 % in Western Europe, while it increased by 57 % during the same time period in the Middle East and Africa [1]. Worst hit by the influence of Tobacco Industries in these countries are the adolescents who are most susceptible to tobacco initiation and sustenance of it thereof. It has been estimated that in LMICs, between 68,000 and 84,000 young people smoke cigarettes each day and are at risk of eventually become long-term smokers [2]. In Nigeria, smoking prevalence’s varies across many studies probably due to the differing methodologies deployed. A study done in 2009 amongst secondary school students across six geopolitical zones in Nigeria reported a current smoking prevalence of 17.1 % [3]. In contrast, the Global Youth Tobacco Survey (GYTS) conducted in 2008 documented a lower smoking prevalence ranging from 2.6 % – 6.2 % amongst secondary school students aged 13–15years [4]. According to the GYTS, the prevalence of current smokers amongst in-school youths in Ibadan was 3.5 % with a wide confidence interval of 0.9 % – 13.0 % [4]. Concerning smoking initiation, a study conducted in 2013 among school going adolescents in Lagos State revealed that 7.3 % had initiated cigarette smoking [5]. The susceptibility of youths to initiate tobacco smoking makes the worldwide efforts geared towards tobacco control justified. One of the strategies for tobacco control in Nigeria is the mandatory text-only warning. Unfortunately, this may not be working as envisaged. Odeyemi et al. reported in a study amongst secondary school students in Lagos State that although up to 82 % of them had seen warnings against smoking, it had no significant effect on their smoking decision [3].

**Fig. 1:**
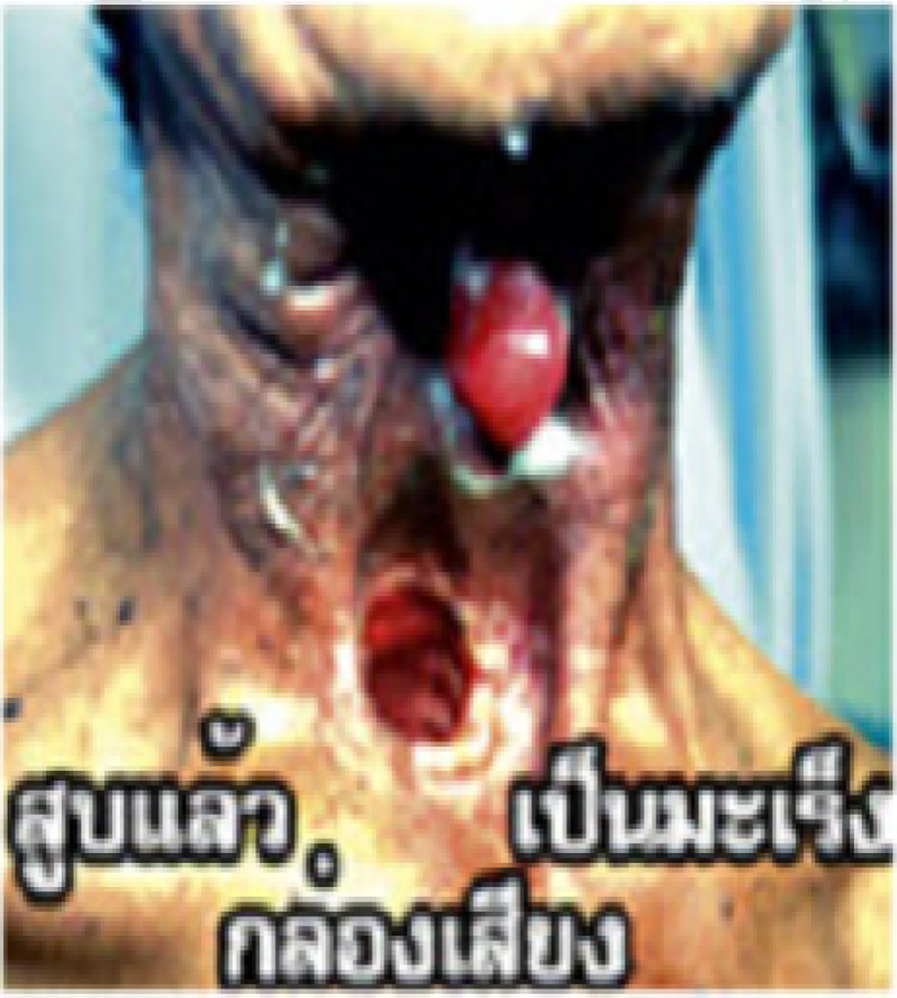
Cigarette smoking causes cancer of the airways

**Fig. 2:**
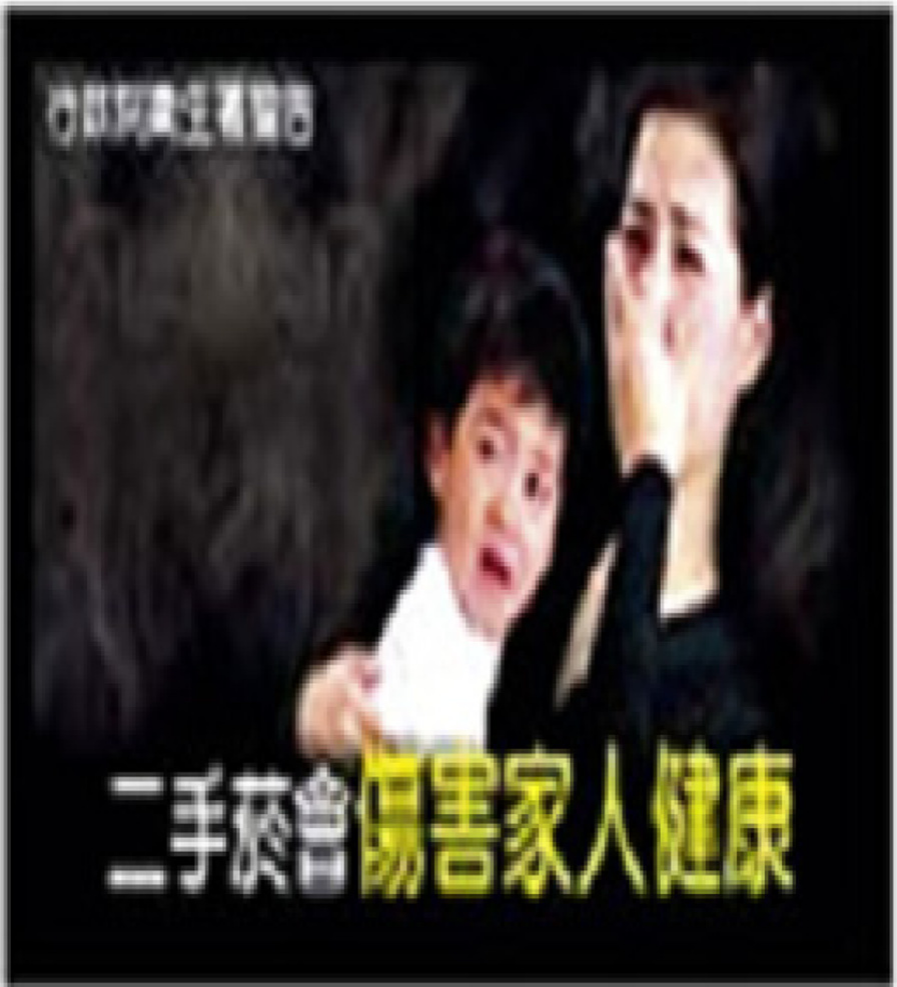
Cigarette smoking harms children

**Fig. 3:**
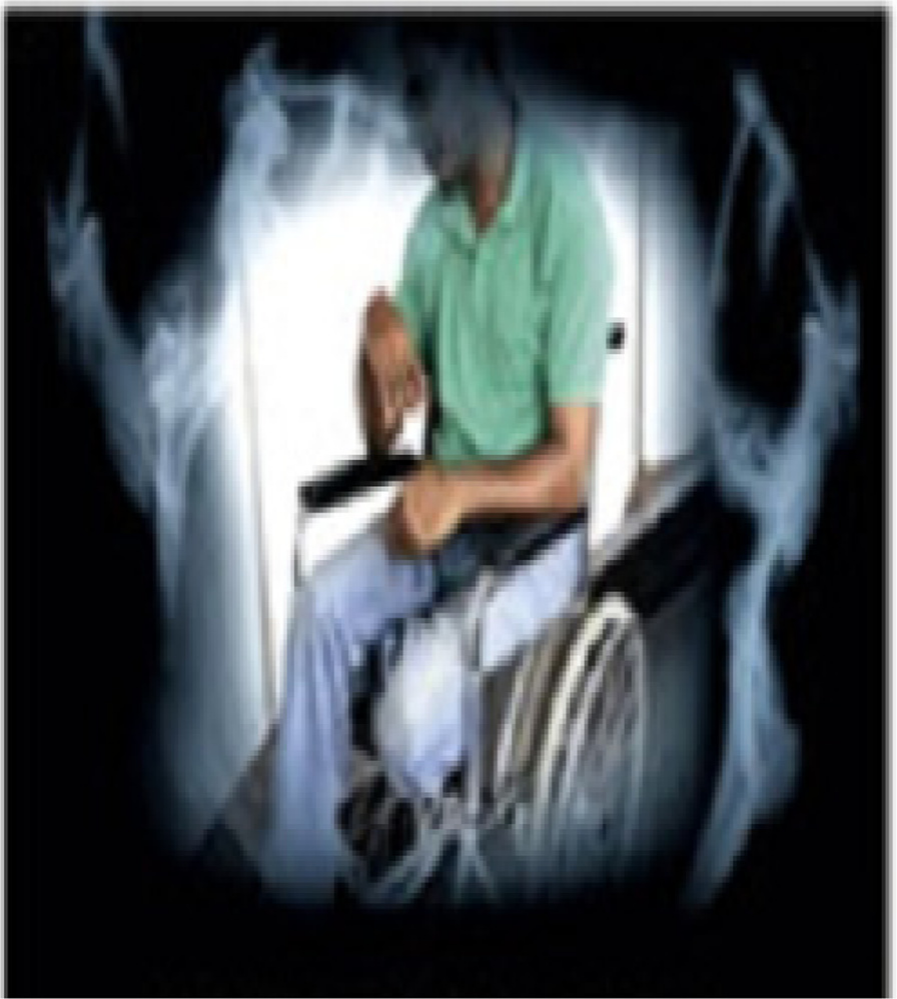
Cigarette smoking causes stroke

**Fig. 4:**
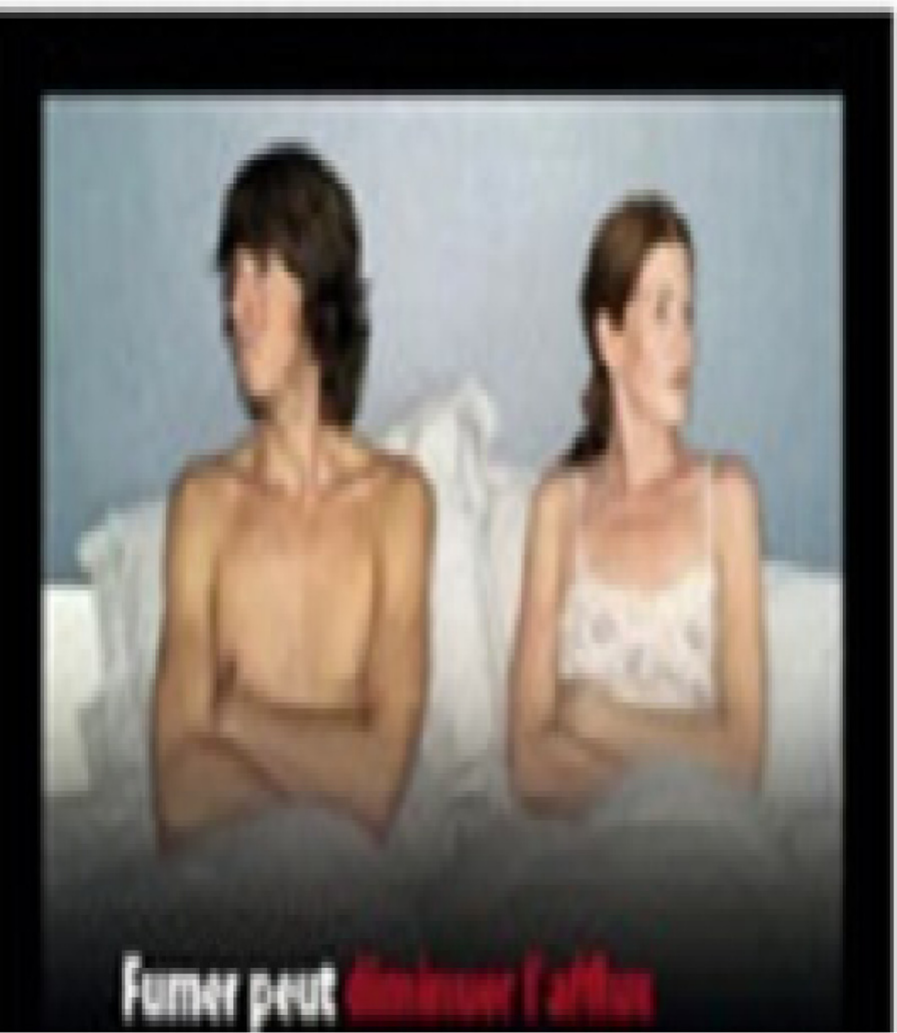
Cigarette smoking causes impotence

According to findings from the International Tobacco Control (ITC) four country surveys, “health warnings on cigarette packages are a prominent source of health information and an effective means of communicating specific disease risks” [6]. Article 11 of the FCTC also presents a unique opportunity for implementing this by making provision for strong, clear and legally obligatory standards for health warning labels on tobacco packaging [7]. Therefore, making the information on the health effects and diseases associated with tobacco use mandatorily available on cigarette packs will be essential in countering the increasing trend of tobacco industry’s deliberate ploy to attract youths to initiate tobacco use.

A review by Hammond reported that health warnings on cigarette packs serve as a powerful source of increasing health knowledge and perception of risks for smokers and nonsmokers. The review went on to conclude that evidence from studies suggests that “pictorial health warnings that elicit strong emotional reactions are significantly more effective” [8]. The desirability of increasing the risk perception of adolescents becomes important against the backdrop of studies showing the apparent gap in understanding the exact nature of the harm posed by tobacco [6, 9].

To increase the effectiveness of packaging and labelling, The FCTC recommends that warnings be designed to target specific subgroups while pre-marketing testing is promoted as an essential method for understanding and assessing the likely effectiveness of any intended graphic warning [7]. To date, Nigeria has not introduced any graphic warning, even though this has been proposed by tobacco control advocates as part of the effort in the on-going process of domesticating FCTC in Nigeria. This study is therefore in-line with the FCTC recommendation to provide local evidence for the enrichment of the advocacy process. In this study, we evaluated the perceived effectiveness of selected graphic warnings on smoking initiation amongst in-school adolescents.

## Methods

This cross-sectional study was conducted amongst secondary school students in Igbo-ora; a semi-rural community in southwest Nigeria. Igbo-Ora is the practice site for the Community and Primary Health Care programme of the University of Ibadan. We used the Leslie Kish formula for single proportion at a 5 % precision level and 95 % confidence interval. We factored in an estimated proportion of 71.6 % (the proportion of participants who reported feeling discouraged to smoke on viewing image) as reported in a study in the United States of America [10]. Thus the calculated sample size was 313. We adjusted for a 10 % non-response rate and design (clustering) effect of 1.5. We eventually interviewed 544 students. Schools were selected using a two-stage sampling technique with the school classes serving as the final sampling unit. Our sampling frame was the list of schools in Igbo-Ora (list of eight schools); out of which two schools were selected. The permission of the school principal was obtained in each of the selected schools. Students in the last three senior secondary levels (SS1, SS2 and SS3) were approached and given a form to take home for their parents’ approval. The last three levels were chosen because the school records showed that most students aged 13–17years were at these levels. Three classes were chosen from each level. Verbal assent was also obtained from the students and all assenting students aged 13–17years were recruited into the study. None of the approached student declined to participate in the study. Recruited students were interviewed using an interviewer assisted pre-tested questionnaire. In each participating class, teachers were excused from the classrooms and each recruited student was given a semi-structured questionnaire. Because the questionnaire was in English language (the official language in Nigeria and not the mother tongue), a research assistant was stationed in each class to clarify any unclear question. However, students filled their own questionnaire independently. The graphic images were introduced one at a time by digital projection with an interval of 5 min apart before students were asked to record their responses in the questionnaire provided. In this article, we report adolescents’ response to graphic health warnings and their perception of the likely effectiveness. Four images; Figs. 1, 2, 3, and 4 (from Thailand, Taiwan, Mauritius and France respectively) were obtained from the pictorial health warning galleries of the World Health Organization and were shown to the respondents’ one after the other [11]. The students were asked to tick on their questionnaire the emotions that the images evoked in them. The emotions assessed were fear, shock, anxiety and indifferent emotion and the proportion of students affected by the various emotions was ascertained for each of the images. Using a three-point Likert scale rating (‘large extent’, ‘little extent’ and ‘no effect’), the students were also asked to rate the likely effect of the images in preventing initiation of smoking.

Bivariate analysis was done to determine the effect of age and gender on the likely effectiveness of the images in preventing smoking initiation. For this analysis, we dichotomized the age group into younger adolescents (‘less than 15 years’) and older adolescents (‘15 years and above’) [12]. Analysis was done with the level of significance set at 5 %.

Ethical approval was obtained from the Ibarapa Programme Research Advisory Committee.

## Results

As shown in Table 1, there were more females 301 (55.3 %) amongst the respondents and students from public secondary schools constituted the majority (89.9 %). A larger proportion were also in Senior Secondary School 1 and 2 (32.4 and 43.0 % respectively).

**Table 1:** Socio-demographic characteristics of respondents

Variable	*n*	%
Sex		
Male	243	44.7
Female	301	55.3
Age*		
< 15 years	302	55.5
≥ 15 years	242	44.5
School		
Public school	456	89.9
Private school	51	10.1
School class		
Senior secondary 1	176	32.4
Senior secondary 2	238	43.8
Senior secondary 3	130	23.9

*mean age: 15.3 ± 1.6 years

Of those interviewed, 40 (7.4 %) indicated that they had ever considered smoking. However, nine (1.7 %) of the students reported having ever smoked; of which two indicated that they were still smokers up till the time of the interview.

Table 2 shows that pictorial image 1 (fig 1) which depicted that “cigarette causes cancer of the airways” evoked fear in 307 (56.4 %) of the respondents. Pictorial image 4 (fig 4) which depicted that “cigarettes causes impotence” evoked fear in 228 (41.9 %) respondents, image 2 (fig 2) depicting that “cigarette smoke harms children” evoked fear in 215 (39.5 %) while image 3 (fig 3) which depicted that “cigarettes causes stroke” evoked fear in 203 (37.3 %) respondents. Similarly, Images 1, 2, 3, and 4 shocked 203 (37.3), 148 (27.2), 179 (32.9) and 127 (23.3 %) respondents respectively. Anxiety was also evoked by the images with 16 (2.9), 115 (21.1), 107 (19.7) and 80 (14.7 %) respondents expressing that they felt this on seeing Images 1, 2, 3 and 4 respectively.

**Table 2:** Emotion evoked by graphic images on consequence of tobacco use

Image	Emotion evoked			
	Fear *n* (%)	Shock *n* (%)	Anxiety *n* (%)	Indifferent *n* (%)
Image 1: Cigarette causes cancer of the airways	307 (56.4)	203 (37.3)	16 (2.9)	18 (3.3)
Image 2: Cigarette smoke harms children	215 (39.5)	148 (27.2)	115 (21.1)	66 (12.1)
Image 3: Cigarettes causes stroke	203 (37.3)	179 (32.9)	107 (19.7)	55 (10.1)
Image 4: Cigarettes causes impotence	228 (41.9)	127 (23.3)	80 (14.7)	109(20.0)

Many of the respondents (76.7 %) perceived that image 1 depicting that cigarette causes cancer of the airways was to a large extent likely to be effective at preventing adolescents from initiating smoking. This perception was also expressed by 243 (44.7), 318 (58.5) and 338 (62.1 %) of respondents to Images 2, 3, and 4 respectively (Table 3).

**Table 3:** Perceived effectiveness of graphic images in preventing adolescents from initiating smoking

	Large extent *n* (%)	Little extent *n* (%)	No effect *n* (%)
Image 1: Cigarette causes cancer of the airways	417 (76.7)	85 (15.6)	42 (7.7)
Image 2: Cigarette smoke harms children	243 (44.7)	229 (42.1)	72 (13.2)
Image 3: Cigarettes causes stroke	318 (58.5)	152 (27.9)	74 (13.6)
Image 4: Cigarettes causes impotence	338 (62.1)	127 (23.3)	79 (14.5)

As seen in Table 4, a significantly higher proportion of students less than 15 years (80.1 % perceived the images that depict that cigarette causes cancer of the airways was to a large extent, probably effective in preventing smoking initiation compared to those who were 15 years and older (72.3 %). The difference was statistically significant at *p* = 0. 032 Similarly, students less than 15 years (66.2 %) significantly perceived image 4 from France depicting that cigarette causes impotence as being to a large extent, probably effective in preventing smoking initiation compared to 57 % who were 15 years and older (*p* = 0.03). However, gender did not significantly influence the respondents’ perception of the likely effectiveness of any of the graphic health warnings.

**Table 4:** The effect of age and gender on perceived effectiveness of graphic health warnings in preventing adolescents from initiating smoking by age and gender

Variable	Perceived effectiveness of image		
	Large extent	Little or no effect	
1. Image of airway cancer			
Age			
< 15 years	242 (80.1)	60 (19.9)	0.032
≥ 15 years	175 (72.3)	67 (27.7)	
Sex			
Male	183 (75.3)	60 (24.7)	
Female	234 (77.7)	67 (22.3)	0.50
2. Image of harm to children			
Age			
< 15 years	138 (45.7)	164 (54.3)	
≥ 15 years	105 (43.4)	137 (56.6)	0.59
Sex			
Male	110 (45.3)	133 (54.7)	
Female	133 (44.2)	168 (55.8)	0.80
3. Image of stroke			
Age			
< 15 years	182 (60.3)	120 (39.7)	
≥ 15 years	136 (56.2)	106 (43.8)	0.39
Gender			
Male	146 (60.1)	97 (39.9)	
Female	172 (57.1)	129 (42.9)	0.49
4. Image of impotence			
Age			
< 15 years	200 (66.2)	102 (33.8)	
≥ 15 years	138 (57.0)	104 (43.0)	
Gender			
Male	157 (64.6)	86 (35.4)	
Female	181 (60.1)	120 (39.9)	0.29

## Discussion

The use of graphic images engages the viewers and enables more effective processing of the information presented with the images [13, 14]. A study conducted among secondary school students before and after the introduction of graphical warnings in Australia documented that school children were more likely to be engaged by and talk about health warnings after graphic warnings were introduced in 2006 [14]. The study also reported that there was increased consideration to quit smoking by the students [14]. The powerful potential effect of graphic images perhaps justifies the huge resources invested by the tobacco industries to maintain brand images as a marketing strategy to enhance consumer retention [15]. This study, which is the first in Nigeria to provide an insight into the likely effectiveness of graphic warnings (if introduced) on tobacco initiation among adolescents shows that more than half of the study participants were afraid of smoking on viewing the image of cancer of the airways. This same image elicited a shock effect on most of the respondents. It is therefore not surprising that more than three quarters of the respondents perceived that this image would probably prevent smoking initiation. This finding aligns with David Hammond’s position in a review on the effect of health messages; in which he posited that vivid fear-arousing pictorials on health consequences of smoking are effective amongst both smokers and non-smokers [8]. Furthermore, Fong and colleagues reported that graphic warnings enhance the effectiveness of warning labels as they could be used to change the demand for tobacco products by using pictorials showing the negative impacts of smoking. This they stated would counter the use of pictorials which the tobacco industries have used for many years to promote smoking as a positive attribute [16]. The emotions the images in this study elicited in the students may be expected to linger in their memory and may create dissonance against influences that aim to promote smoking initiation among them.

The images that respondents were least afraid of were those depicting the effect of cigarettes on stroke causation and harm to children. This finding is very important, especially within the context of countries that are yet to introduce graphic warning labels. Young people may be minimally engaged and unable to relate appropriately to issues that are not perceived as immediate threats to them. For instance, stroke may be seen as a remote issue because of the low prevalence of hypertension amongst adolescents. Studies amongst asymptomatic populations of secondary school students have documented low prevalences’ of between 3.2 and 5.4 % [17, 18]. Furthermore, adolescents may also perceive stroke to be a disease of adults. Similarly the idea of having children may seem distant to them in their present state. This is one of the reasons why FCTC recommends that warnings may be designed to target specific sub-groups [7].

Also, the image depicting that cigarette smoking can cause impotence evoked much fearful emotion in the respondents as slightly more than two-fifths of them expressed fear. Close to two-thirds of respondents felt that it would likely get people to quit smoking or stop its initiation. The masculinity and virility discourse has been around the African continent for a long time and most times are routed in cultural ethos. Adolescents are part of society and are often under extreme pressure to take up the dominant discourse. A sexual health needs assessment conducted amongst young people aged 13–19years in Uganda documented that *“all participants felt that young people begin their sexual lives too early and young men feel under pressure from friends and older men to prove their masculinity” *[19]. It is against this dominant discourse that our respondents’ views about the likely effectiveness become logical.

Our study did not demonstrate any association of effectiveness with gender and only with the images on cancer of the airway and impotence was there an age differential. This differential may have been due to more respondents expressing that these images evokes fear in them, especially as age is also known to determine fear. This differs slightly from the finding by Fong et al. that the emotions graphic warnings evoke in people are likely to apply across age groups and gender [20].

## Conclusion

Our study has provided a very useful insight into the perceived effectiveness of graphic health warnings and the emotions it is likely to evoke in adolescents. Our findings suggest that pictorial health warnings that evoke fear may likely influence the decision to initiate smoking of cigarettes by adolescents. These emotions may ensure improved memory and salience for messages depicting the effects of cigarettes. We posit that this provides a useful starting point and platform for exploring the effectiveness of graphic warnings in adolescents and other populations in Nigeria. Currently this has become more important as the Nigerian tobacco control act (with provisions for graphic health warnings) became operational (2014) after this study was conducted.

## Consent

Written informed consent was obtained from the students parents/guardians for the conduct of the study while verbal assent was obtained from individual students.
